# Osteoconductive Silk Fibroin Binders for Bone Repair in Alveolar Cleft Palate: Fabrication, Structure, Properties, and In Vitro Testing

**DOI:** 10.3390/jfb13020080

**Published:** 2022-06-14

**Authors:** Supaporn Sangkert, Kantida Juncheed, Jirut Meesane

**Affiliations:** Institute of Biomedical Engineering, Faculty of Medicine, Prince of Songkla University, Hat Yai 90110, Songkhla, Thailand; dek-supya@hotmail.com (S.S.); kantida.juncheed@gmail.com (K.J.)

**Keywords:** silk fibroin, binder, bone repair, maxillofacial surgery

## Abstract

Osteoconductive silk fibroin (SF) binders were fabricated for the bone repair of an alveolar cleft defect. Binders were prefigureared by mixing different ratios of a mixture of random coils and SF aggregation with SF fibrils: 100:0 (SFB100), 75:25 (SFB75), 50:50 (SFB50), 25:75 (SFB25), and 0:100 (SFB0). The gelation, molecular organization, structures, topography, and morphology of the binders were characterized and observed. Their physical, mechanical, and biological properties were tested. The SF binders showed gelation via self-assembly of SF aggregation and fibrillation. SFB75, SFB50, and SFB25 had molecular formation via the amide groups and showed more structural stability than SFB100. The morphology of SFB0 demonstrated the largest pore size. SFB0 showed a lowest hydrophilicity. SFB100 showed the highest SF release. SFB25 had the highest maximum load. SFB50 exhibited the lowest elongation at break. Binders with SF fibrils showed more cell viability and higher cell proliferation, ALP activity, calcium deposition, and protein synthesis than without SF fibrils. Finally, the results were deduced: SFB25 demonstrated suitable performance that is promising for the bone repair of an alveolar cleft defect.

## 1. Introduction

Currently, many individuals are born with an alveolar cleft palate that results from the malformation of the oral cavity during fetal development [1]. There are three types of cleft palate: (1) incomplete, (2) complete, and (3) bilateral. For severe cases, the defect includes a large area of the soft and bone tissue. In cases of a large area, the bone defect leads to irregular speech and feeding. Many patients need surgery with or without biomaterials for bone repair of the alveolar cleft palate [2]. The need for biomaterials depends on the severity of the defect size. For a small defect size, surgeons generally operate without biomaterials, using a technique of flapping to repair the tissue at the defect site and recover the contour shape around the oral area. On the other hand, a large defect size that includes the oral mucoperiosteum and bone may require surgery with biomaterials, as a supplement to promote tissue repair to reconstruct the contour shape [3]. In particular, a bone-defect area requires biomaterials when grafting. Some biomaterials for bone grafting include hydroxyapatite, β-tricalcium phosphate, biphasic calcium phosphate, and calcium sulphate [4]. These materials show good performance to promote new tissue formation. Nevertheless, in some cases, inorganic compounds are used as grafting materials for bone repair [5]. However, these compounds are available as powders that are difficult to mold before implantation into a bone defect [6]. Furthermore, research has demonstrated that some inorganic compounds are super-stiff [7,8], which leads to irregular bone formation at the interfacial area between the compounds and the tissues [9].

The surgical bone repair of an alveolar cleft palate requires the use of grafting materials to recover the contour shape at the oral area and promote new tissue formation [10]. Hydroxyapatite (HA), tri-calcium phosphate, biphasic calcium phosphate, and calcium sulphate are examples of ceramic compounds for bone grafting in bone repair [11]. These materials are normally powders placed into the defect space. The drawback of powders is the difficulty of molding to fit the defect area. The powder only physically attaches to the bone, which leads to insufficient physical and mechanical stability to maintain and recover the regular contour of the shape of the oral cavity. Furthermore, the powders exhibit hyper-stiffness that can cause irregular cell response, leading to malformation of the tissue at the interface area. Therefore, the powders need adhesive binders to enhance the stability of molding and reduce the hyper-stiffness of the powders [12]. Some studies have proposed mixing a polymer-based binder with those compounds to reduce the super-stiffness [13,14]. This form of binder is adhesive and is compatible with ceramic compounds and bone tissue [15]. The binder is physically stable, which is suitable to support ceramic compounds to maintain the shape after implantation into the defect space, without collapsing during new bone formation [16]. Furthermore, some studies report mixing a ceramic compound with a binder that exhibits mechanical properties similar to natural bone, which is able to promote regular new bone formation at the interface area between the compound and tissue.

Currently, several kinds of binders are available that are either natural materials or synthetic materials, which have been used for the bone repair of an alveolar cleft defect. Collagen, for example, is a natural binder for bone repair [17]. Natural binders have the advantages of biocompatibility and non-toxicity. Nevertheless, natural binders degrade rapidly, which does not match the growth rate of new tissue formation and leads to the collapse of the contour shape during tissue repair. Many synthetic binders are available, such as calcium sulfate and carboxymethylcellulose [18]. Synthetic binders have good stable mechanical properties that are able to maintain the contour shape without collapse during tissue repair. However, some synthetic binders degrade slowly, which leads to irregular tissue formation at the surface. Therefore, osteoconduction is a bioactive function of high-performance binders that is needed for bone healing [19]. However, few studies have focused on the osteoconductive function of binders [20,21]. Therefore, our research aimed to fabricate novel binders that exhibit osteoconductivity, for the repair of alveolar cleft defects.

Silk fibroin (SF) is a natural protein with good mechanical properties of high strength and toughness [22], which has good biocompatibility [23] and osteoconductivity for bone formation [24,25,26]. The molecular structure of SF is in three forms: (1) random coil, (2) alpha helix, and (3) beta sheet. Each structural form shows different properties. For instance, random coil has a looser structure than alpha helix and beta sheet. Therefore, it is a more flexible material than the others. On the other hand, beta sheet has dense packing via hydrogen bonding, which offers higher mechanical strength and toughness than the others. In the bone repair of an alveolar cleft defect, SF has been prepared into two- and three-dimensional biomaterials that are inserted into the defect area to recover the contour shape of the oral cavity [27]. Some studies have incorporated SF with other materials, to improve the enhancement of bone formation suitable for an alveolar cleft defect [28]. However, studies using SF as a binder are rare [29]. In this research, SF was selected as the base material to fabricate a binder with osteoconductive properties for the bone repair of an alveolar cleft defect.

The osteoconductive binders were prepared as mixtures of random coils and SF aggregation with SF fibrils [30]. The binders were then characterized for their molecular formation, structural organization, and morphology. Following the characterization of the binders, in vitro testing was performed to demonstrate their potential osteoconductive properties. Finally, the binders were evaluated for their potential in the bone repair of an alveolar cleft defect.

## 2. Materials and Methods

### 2.1. Preparation of the Silk Fibroin Solution

*Bombyx mori* silk cocoons were cut into small pieces and boiled for 30 min in 0.02 M Na_2_CO_3_ two times to remove the sericin, followed by washing with dH_2_O, and then they were kept at 60 °C until dry. The SF was dissolved in 9.3 M LiBr for 4 h at 70 °C. The dissolved SF was then poured into a dialysis membrane tube (3500 MWCO), to remove LiBr against dH_2_O for about 72 h. The SF solution was centrifuged at 3500× *g* rpm for 30 min to remove debris and stored at 4 °C until use.

### 2.2. Extraction of a Mixture of Random Coil and Aggregation of Silk Fibroin

A 5% (*w*/*v*) SF solution was used for silk-film preparation. Aliquots of 2.7 mL of the SF solution were poured into 3 × 3 cm plastic molds for 24 h at 25 °C until dried films were obtained. Afterward, the soluble parts were extracted from the SF films, which were then cut into small pieces and immersed in dH_2_O for 1 h at 37 °C. To separate the mixture of random coils and aggregated SF, the SF film in the dH_2_O was centrifuged at 3500× *g* rpm for 15 min. This mixture showed a concentration of 0.3% (*w*/*v*). It was kept in a refrigerator until use [31].

### 2.3. Preparation of the Silk Fibroin Fibrils

The SF solution was prepared following the procedure by Ling et al. [32]. The 5% (*w*/*v*) SF solution was diluted with dH_2_O to give a final concentration of about 0.3% *w*/*v*. This SF solution was kept at 60 °C for 120 h, and the SF fibril suspension was obtained in this process. The SF fibril suspension was refrigerated until use.

### 2.4. Preparation of Binders

The binders were prepared by combining the mixture of random coils and aggregation of SF with the SF fibrils in ratios of *v*/*v* (Table 1).

### 2.5. Analysis of Self-Assembly during Gelation of Binder

After preparation of the binders following the ratios in each group, 200 µL of each binder was added into 96-well plates and incubated at 37 °C. Time points from 0 min to 120 min were selected for observation of gelation. Self-assembly during gelation of the binder was estimated using a microplate spectrophotometer (Thermo Fisher Scientific, Vantaa, Finland) at a wavelength of 310 nm.

### 2.6. Fourier Transform Infrared (FTIR) Spectroscopy

Samples of the binders listed in Table 1 were poured into plastic molds to obtain the film coatings and then kept at 37 °C until dry. The dried binders were then removed from the plastic molds. The molecular organization of each binder was analyzed by FTIR using KBr pellets in a FTIR spectrophotometer (EQUINOX 55, Bruker Optics, Ettlingen, Germany) in the range of 50–4000 cm^−1^ for the analysis.

### 2.7. Differential-Scanning Calorimetry (DSC)

After the dried binders were kept in a desiccator for 24 h, they were cut into small pieces, put in an aluminum pan, and covered. The weight of each sample was recorded for data analysis, and the characterization was set by a DSC instrument (DSC7, Perkin Elmer, Waltham, MA, USA). The temperature was set at a range from 20 °C to 350 °C.

### 2.8. Topography and Surface Roughness Analysis

The binders from each group were dropped on a glass slide for gelation for 1 h at 37 °C, which was then followed by drying at 25 °C for 24 h. The topography of the samples was observed under a Nikon Inverted Microscope Eclipse Ti-S. Microscopy images were used to calculate the surface roughness of the SF binders using the ImageJ program [33].

### 2.9. Scanning Electron Microscopy (SEM)

Samples of the binders were poured into 48-well plates before freeze drying. The freeze-dried binders were fixed on an SEM stub and coated with gold by a gold-sputter-coating machine (SPI Supplies, Structure Probe Inc., Westchester, PA, USA). The morphologies of the samples were then observed using an SEM machine (Quanta 400, FEI, Brno, Czech Republic).

### 2.10. SF Release

The binder from each group was coated on 48-well plates and left to dry at room temperature for about 24 h. Into each well, dH_2_O was added to monitor SF release from the binder at different time points: 0.5 h, 1 h, 2 h, 4 h, 8 h, 12 h, and 24 h. At each time point, the solution was collected, and the protein release was measured in accordance with the instructions of the manufacturer for the Pierce™ BCA Protein Assay Kit (Thermo Fisher Scientific, Waltham, MA, USA). The total protein (µg) was calculated according to the standard protein curve, and the release of proteins at each time point of 0.5 h was added with the next time point until 24 h was reached completely for the cumulative plot of the release [34].

### 2.11. Water Contact Angle

Dried binders were placed on glass slides and tested with a water-contact-angle machine (Optical Contact Angle Analyzer, OCA25; Data Physics Instruments GmbH, Filderstadt, Germany). The WI-RES-Contact angle-001 and REF-RES-Contact angle-001 settings were used for standard testing, which was accomplished by dropping 50 µL of dH_2_O on the gel coating of the glass slide, and the angle was measured.

### 2.12. Testing the Binding Function as a Mechanical Property

The binding function of the binders in each group was observed by using a Lloyd Instruments testing machine. A glass slide was cut into a rectangular shape and 400 µL of SF binder was dropped on the glass slide, and another glass slide covered the SF binder in an overlapping manner. The SF binder was formed under 37 °C for 1 h and allowed to dry at room temperature before testing. The speed compression was set at 1.00 mm/min. The testing and data were collected until the glass slide became detached.

### 2.13. Cell Culturing

The MC3T3E1 osteoblast cell line was used to evaluate the biocompatibility and differentiation of all the dried SF binders. The cells were seeded on the dried SF binder at a density of 1 × 10^5^ cells and cultured in alpha-MEM medium (α-MEM; Gibco, Invitrogen, Carlsbad, CA, USA) with the addition 10% fetal bovine serum, 1% penicillin/streptomycin, and 0.1% Fungizone at 37 °C in a humidified 5% CO_2_/95% air incubator. The media were changed every 3–4 days during culturing. In the differentiation stage, an osteogenic medium (OS) containing 20 mM b-glycerophosphate, 50 µM ascorbic acid, and 100 nM dexamethasone (Sigma-Aldrich, St. Louis, MO, USA) was used for culturing.

### 2.14. Cell Proliferation

Cell proliferation on the SF binders was detected with PrestoBlue^®^ Cell Viability Reagent (Invitrogen). The PrestoBlue^®^ reagent was mixed with the media at a ratio of 1 to 10 before being added to the cells and then incubated for 1 h. A sample aliquot of 200 µL was removed from the cell mixture and added to 96-well plates for optical-density (OD) measurement at a wavelength of 600 nm. After testing with the PrestoBlue^®^ reagent, the cell mixtures were washed with phosphate-buffered solution (PBS) and cultured for cell-proliferation measurements from day 1 to day 7.

### 2.15. Cell Viability

Cell viability was assessed with fluorescein diacetate (FDA) at days 1, 3, 5, and 7. FDA was dissolved in acetone at a concentration of 5 mg/mL. Before staining, the cells were washed twice with PBS. Next, fresh media was added onto the SF binder films with 5 µL of FDA solution and incubated for 5 min. The SF binder films were washed 2–3 times with PBS and examined under fluorescence microscopy [35].

### 2.16. Protein Synthesis

After cell culturing on days 7, 14, and 21 under OS conditions, the osteoblast cells were washed with PBS two times. Next, 800 µL of lysis solution (1% Triton X-100 in PBS) was added to each well, and the wells were kept at −80 °C for 1 h and then returned to room temperature for 1 h. This sequence was repeated for three cycles. The cell-lysis solution was analyzed using the Pierce™ BCA Protein Assay Kit in accordance with the instructions of the manufacturer (Thermo Fisher Scientific, Waltham, MA, USA).

### 2.17. Alkaline Phosphatase (ALP) Measurement

The cell-lysis solutions were used for alkaline-phosphatase (ALP) measurements. An aliquot of 20 µL of cell-lysis solution was tested using an ALP-detection kit (Abcam^®^, Cambridge, UK). The ALP was observed at an OD of 405 nm.

### 2.18. Alizarin Red Staining

After cell culturing for 7 days and 14 days, the cells were fixed with 4% formaldehyde after washing several times with PBS. The cells were stained with 2% Alizarin red for 15 min under ‘avoiding the light’ conditions. The cells were then washed with dH_2_O, and the calcium nodules were assessed under a Nikon Inverted Microscope Eclipse Ti-S.

### 2.19. Statistical Analysis

The testing of five samples in each binder included protein release, stability, cell proliferation, protein synthesis, and ALP activity. The results were statistically compared by one-way analysis of variance (ANOVA) and Tukey’s honestly significant difference test. Statistical significance was set at *p* < 0.05.

## 3. Results

### 3.1. Gelation of the Silk Fibroin Binders

**Figure 1:**
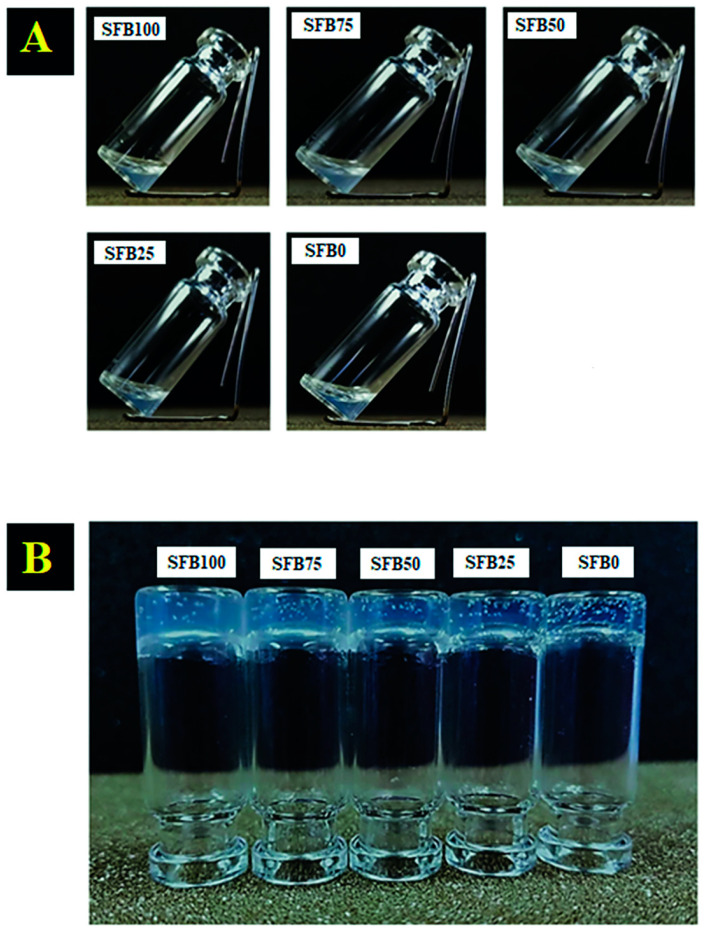
Mixtures of random coils and SF aggregation with SF fibrils: (**A**) before incubation; (**B**) after incubation at 37 °C for 30 min.

Gelation of all SF binders was observed after 30 min at 37 °C. Good gel formation was found in all binders (Figure 1A,B). The ratio of the mixture of random coils and SF aggregation to SF fibrils affected the color of the binders, and turbidity was found in all binders.

**Figure 2:**
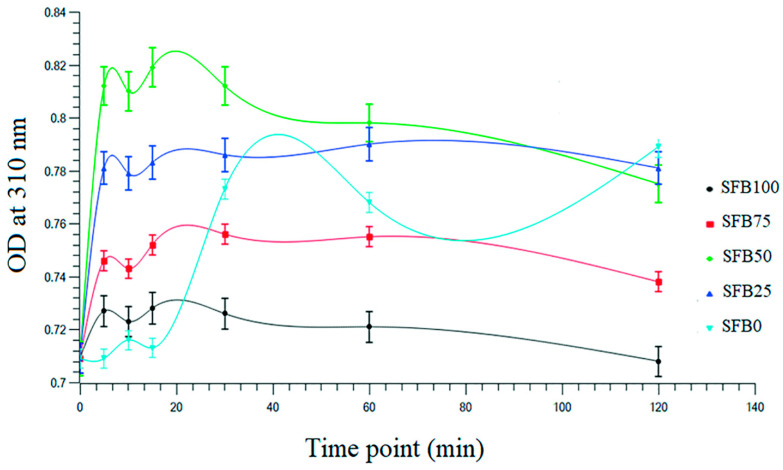
Kinetics of gelation of the SF binders.

To analyze the details of gelation, the SF binders were characterized by a microplate reader at different time points. All data were then plotted to demonstrate the kinetics of gelation. The graph showed two broad peaks. The SFB100, SFB75, SFB50, and SFB25 binders demonstrated the first peaks at around 5 min and the second peaks exhibited at around 20 min (Figure 2). All of the SFB100, SFB75, SFB50, and SFB25 binders had similar patterns. However, the SFB0 binder showed its first peak at around 10 min, and the second peak was exhibited at around 40 min. The SFB0 binder demonstrated broader peaks than the others.

**Figure 3:**
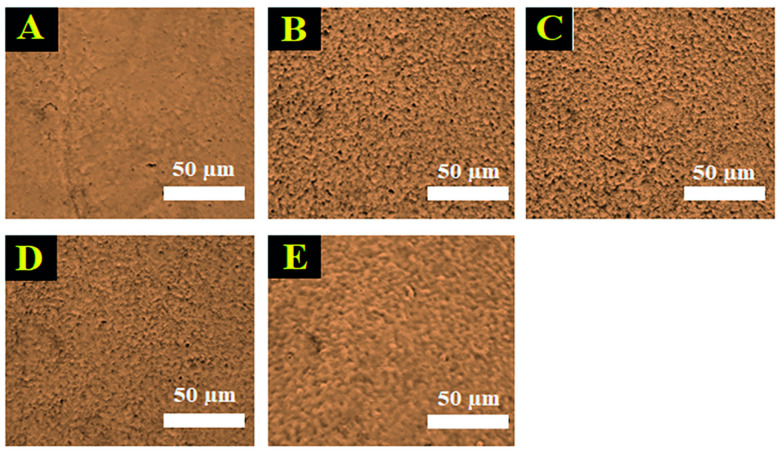
Surface topographies of the SF binders after coating on glass slide from light microscopy: (**A**) SFB100; (**B**) SFB75; (**C**) SFB50; (**D**) SFB25; (**E**) SFB0.

To observe the surface topographies of the binders, all samples were prepared as coated-hydrogel films on glass slides. The topographies of the binders were observed under a light microscope after fixing and drying on glass slides. The SFB100 binder demonstrated a smoother surface compared with the others (Figure 3). The SFB75 and SFB50 binders had rougher surfaces, and the films were porous throughout. A combination of rough and smooth structures were found in the SFB25 and SFB0 binders.

**Figure 4:**
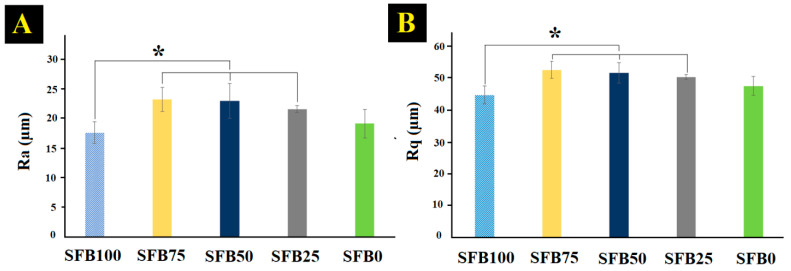
Surface roughness of SF binders: (**A**) arithmetical mean roughness (R_a_); (**B**) root mean square roughness (R_q_) (* *p* < 0.05).

The surface roughness of the SF binders in all groups was expressed as arithmetical mean roughness (R_a_) and root mean square roughness (R_q_) (Figure 4). The R_a_ of the SFB100 binder was lower compared with the others. The combination of random coils and SF aggregation/SF fibrils promoted the formation of a rough surface, and as the ratio of the random coils and SF aggregation decreased, the R_a_ decreased and equaled the R_q_ value.

**Figure 5:**
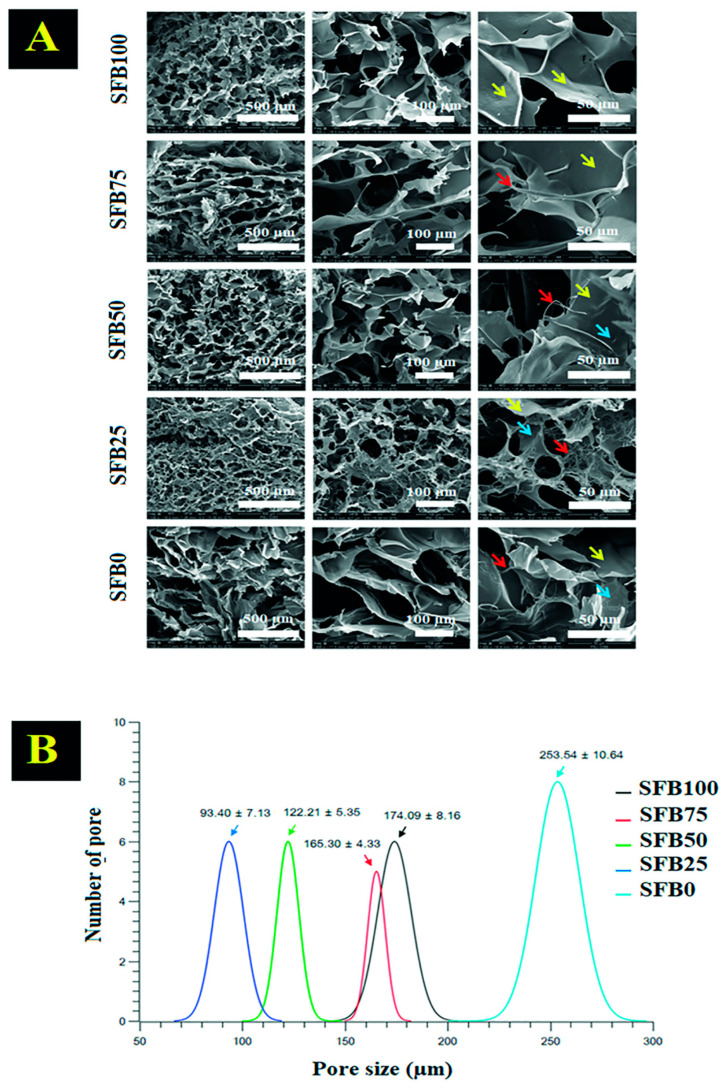
(**A**) SF binder morphology after freeze drying, observed under SEM (red arrows indicate SF fibrils, yellow arrows indicate a smooth surface, and blue arrows indicate embedded SF fibrils in the surface); (**B**) pore size distribution of the binders after freeze-drying.

The morphologies of the freeze-dried binders were observed under SEM [36]. Our results demonstrated that the morphologies were quite different for each of the different mixture ratios of random coils and SF aggregation/SF fibrils (Figure 5A). The SFB100 binder demonstrated interconnected porosity with a consistent pore size and a smooth surface. As the ratio of mixture of random coils and SF aggregation/SF fibrils increased, the pore size became smaller than the SFB100 binder, and the porous structure was not as regular in the SFB75 binder. The surface of the SFB75 binder was smooth, and some fibrils had infiltrated the morphological structure. The SFB50 binder showed a rougher surface, with fibrils embedded in the morphology of the porous-surface layer. A clear fibril-structure formation was found in the SFB25 binder, and the fibrils were found throughout the morphology. Furthermore, the morphological structure showed sub-microporosity, which exhibited pore interconnectivity.

The pore size distribution was examined by SEM, and the pore sizes were measured using the ImageJ program. The SFB0, SFB25, SBF50, SBF75, and SBF100 binders showed pore sizes of 253.54 ± 10.64, 93.40 ± 7.13, 122.21 ± 5.35, 165.30 ± 4.33, and 174.09 ± 8.16 µm, respectively. The SFB100 binder had a larger pore size than the SFB75, SFB50, and SFB25 binders. Higher amounts of SF fibrils in the binder led to smaller pore sizes. However, the SFB0 binder had the largest pore size (Figure 5B).

### 3.2. FTIR and DSC Characterization, Contact Angle, and Silk Fibroin Release

**Figure 6:**
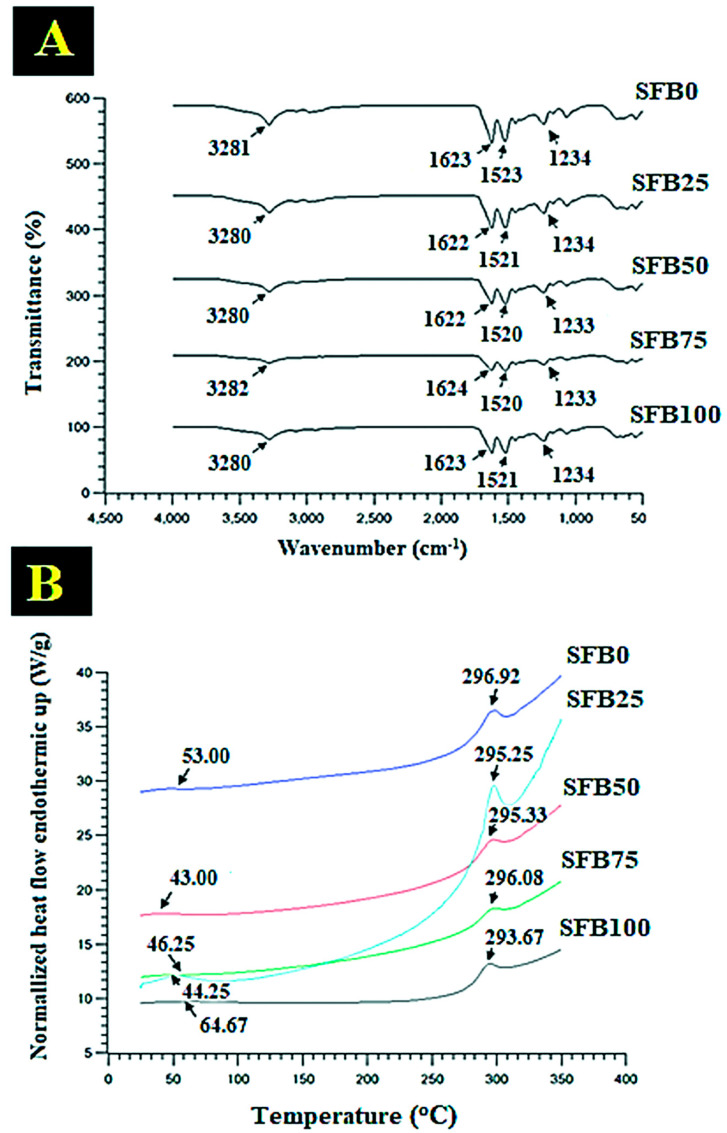
Test results of all binders: (**A**) FTIR spectra; (**B**) DSC thermograms.

The amide groups of the SF were observed with FTIR spectra (Figure 6A). The amine group of the SF appeared at around 3400–3500 °C [37]. A peptide backbone of amide I (C=O stretching) was found at 1260 cm^−1^, with the characteristic vibration bands [38]. The amide II (N-H bending) was observed at 1520 cm^−1^ [39]. Amide III was shown to peak at 1230 cm^−1^ and 1444 cm^−1^ (C-N stretching) [40]. A peak at 694 cm^−1^ was formed for amide IV [41]. The β-sheet structure was indicated by the shift of the absorption peaks between 1622–1624 cm^−1^ (amide I), 1520–1523 cm^−1^ (amide II), and 1233–1234 cm^−1^ (amide III) [42]. The results demonstrated that SFB0, SFB25, SBF50, SBF75, and SBF100 binders had wavenumbers of the amine groups at 3281 cm^−1^, 3280 cm^−1^, 3280 cm^−1^, 3282 cm^−1^, and 3280 cm^−1^, respectively. of the SFB0, SFB25, SBF50, SBF75, and SBF100 binders showed at 1623 cm^−1^, 1622 cm^−1^, 1622 cm^−1^, 1624 cm^−1^, and 1623 cm^−1^, respectively. The SFB0, SFB25, SBF50, SBF75, and SBF10 binders for amide II showed at 1523 cm^−1^, 1521 cm^−1^, 1520 cm^−1^, 1520 cm^−1^, and 1521 cm^−1^, respectively. The SFB0, SFB25, SBF50, SBF75, and SBF100 binders for amide III showed at 1234 cm^−1^, 1234 cm^−1^, 1233 cm^−1^, 1233 cm^−1^, and 1234 cm^−1^, respectively.

Thermal behavior was observed from 25 °C to 350 °C (Figure 6B). The first peak was in the region of 43.33–64.67 °C, which represented the residual water in the SF [43]. The second peak was in the region of 293.67–296.02 °C, which represented the thermal degradation of the SF [44]. The first peak of the SFB0, SFB25, SBF50, SBF75, and SBF100 binders showed residual water in the SF at 53.00 °C, 43.00 °C, 46.25 °C, 44.25 °C, and 64.67 °C, respectively. The second peak of the SFB0, SFB25, SBF50, SBF75, and SBF10 binders was at the degradation temperature of SF, which occurred at 296.92 °C, 295.25 °C, 295.33 °C, 296.08 °C, and 293.67 °C, respectively. The SFB100 binder had the lowest thermal peak at 293.67 °C, and the SFB0 binder had the highest degradation temperature at 296.92 °C.

**Figure 7:**
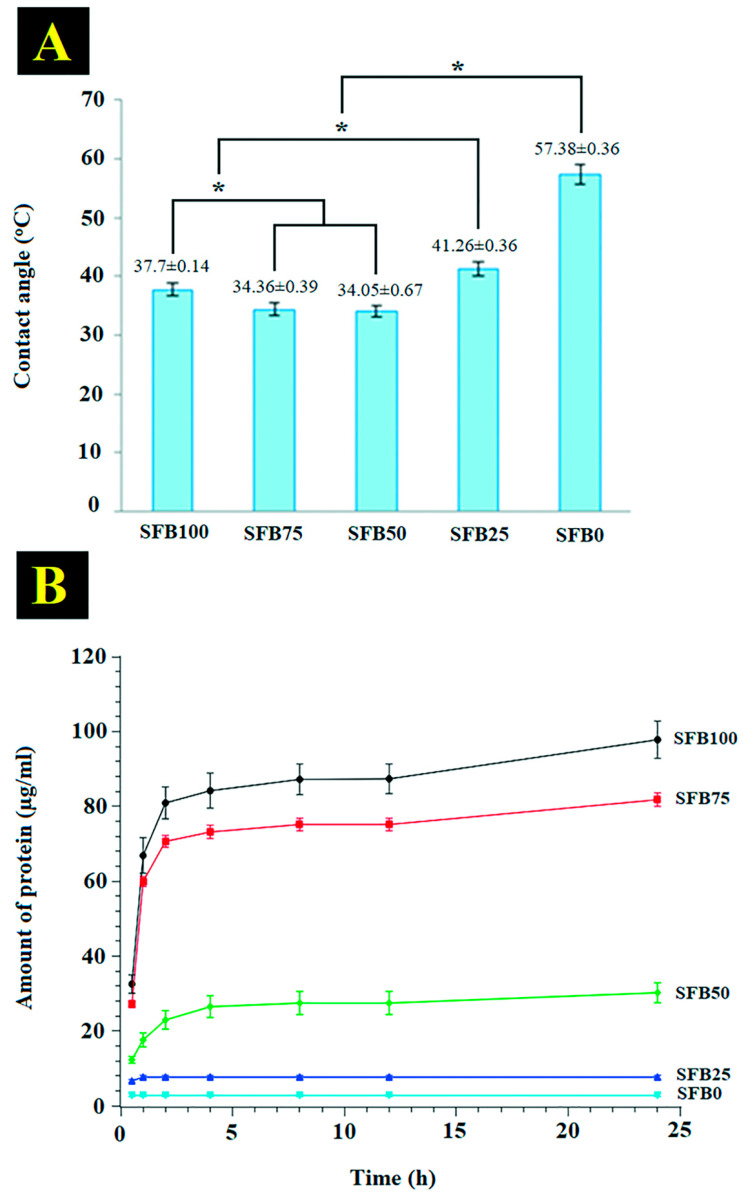
(**A**) Water-contact-angle measurements of all binders coated on glass slides; (**B**) protein release from all binders coated on 48-well plates (* *p* < 0.05).

The hydrophilicity of the SF binders was observed by water-contact-angle measurement. The results showed that higher ratios of random coil and SF aggregation to SF fibrils had lower water-contact angles than the lower ratios (Figure 7A). The SFB0, SFB25, SBF50, SBF75, and SBF100 binders had contact angles of 57.38 ± 0.36, 41.26 ± 0.36, 34.05 ± 0.67, 34.36 ± 0.39, and 37.70 ± 0.14, respectively. The contact angle of the SFB50 binder was the lowest, but it was not significantly different than the SFB75 binder. The contact angle of the SFB100 binder was significantly higher than the SFB75 and SFB50 binders, but it was lower than the SFB25 and SFB0 binders. The hydrophilicity values of the SFB100, SFB75, and SFB50 binders were better than the SFB25 and SFB0 binders. The SFB0 binder had the lowest hydrophilicity.

SF release was measured from the binders at different time points (Figure 7B). The SFB100, SFB75, and SFB50 binders started with more burst-release behavior than the SFB25 and SFB0 binders, from 0.5 h to 1 h after incubation. Then, the SFB100, SFB75, and SFB50 binders kept more release behavior than the SFB25 and SFB0 binders., until 24 h. The SFB100, SFB75, and SFB50 binders reached a steady stage of release after 4 h. The SFB25 and SFB0 binders approached to steady stage of release after 1 h. The result showed the SF release of the binders was SFB100 > SFB75 > SFB50 > SFB25 > SFB0 after 24 h. The SFB100 binder had the highest release. On the other hand, the SFB0 binder exhibited the lowest release.

### 3.3. Binding Function as a Mechanical Property

**Figure 8:**
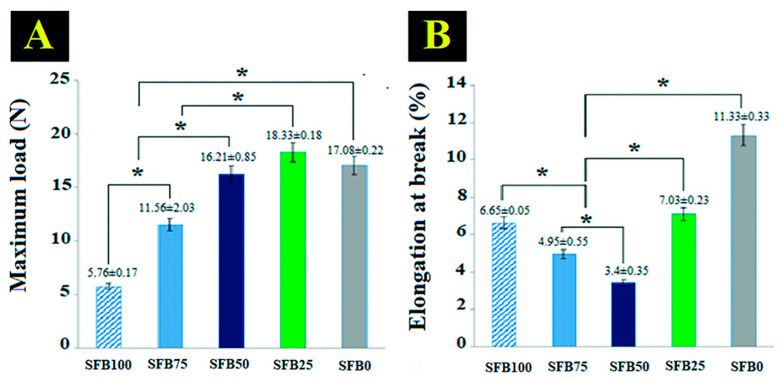
Binding function as a mechanical property of all binders: (**A**) maximum load; (**B**) elongation at break (* *p* < 0.05).

The binding function of the SFB100 binder had the lowest maximum load (Figure 8A). The maximum load of the SF binders increased as the amount of SF fibrils increased. The SFB25 binder exhibited the highest maximum load. The elongation at break of the binders decreased as the amount of SF fibrils decreased (Figure 8B). However, the SFB75 binder had an elongation at break higher than the SFB50 binder. The SFB50 binder had the lowest elongation at break.

### 3.4. Cell Viability and Cell Proliferation

**Figure 9:**
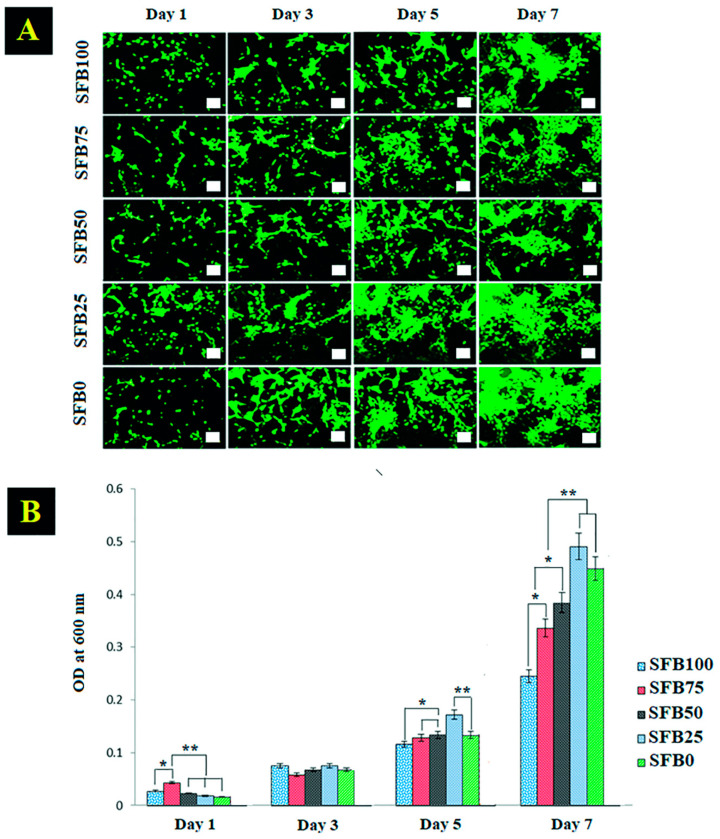
(**A**) Cell viability on days 1, 3, 5, and 7; (**B**) cell proliferation was measured with PrestoBlue on days 1, 3, 5, and 7. Scale bar = 100 µm. (* *p* < 0.05, ** *p* < 0.005).

Cell viability was observed by FDA staining on days 1, 3, 5, and 7. There was sparse cell viability on day 1 that increased on day 3 (Figure 9A). The cell morphologies of the SFB0, SFB25, SFB50, SFB75, and SFB100 binders started to form spindle and elongated shapes on day 3. On day 5, the cells showed dense aggregation, particularly in the SFB25 and SFB0 binders. Cell proliferation was measured on days 1 to 7 with PrestoBlue reagent (Figure 9B). The SFB25 binder showed significantly higher proliferation on days 5 and 7. Cell proliferation was slightly higher from day 1 to day 3. The SFB100 binder showed the highest cell proliferation on day 3. On days 5 and 7, the SFB25 binder had significantly increased cell proliferation than the others. On day 7, the SFB25 binder had the highest cell proliferation, while the SFB100 binder had the lowest. Cell proliferation improved as the amount of SF fibrils increased.

### 3.5. Alizarin Red Staining, Alkaline Phosphatase Activity, and Protein Synthesis

**Figure 10:**
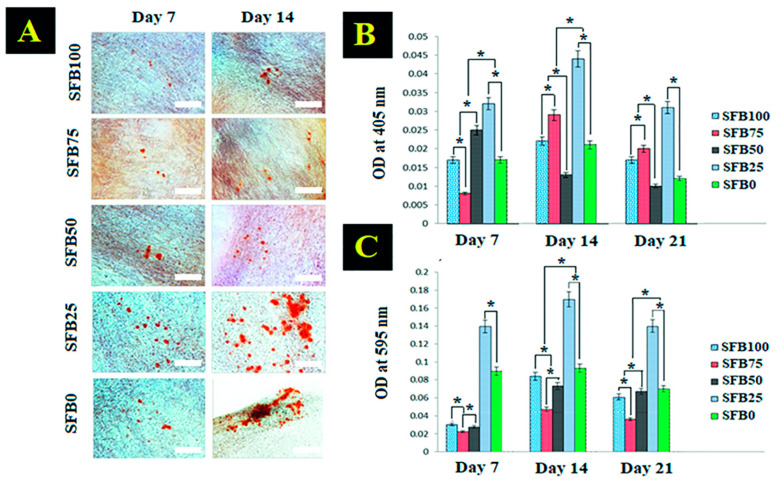
(**A**) Alizarin red staining for calcium-nodule detection on days 7 and 14; (**B**) alkaline phosphatase activity on days 7, 14, and 21; (**C**) protein synthesis on days 7, 14, and 21. Scale bar = 100 µm (* *p* < 0.05).

Calcium nodules were found in all binders using Alizarin red staining (Figure 10A). The SFB100 and SFB75 mixtures showed slight calcium deposition on their surfaces. Overall, calcium deposition was higher as the percentage of SF fibrils increased. The SFB50, SFB25, and SFB0 binders showed more calcium deposition compared with the others on day 7. Calcium deposition clearly increased from day 7 to day 14, especially on the SFB25 and SFB0 binders, which showed larger calcium depositions for nodules with red indicators on the surface.

ALP activity is the osteoblast-phenotype marker in the early stage of bone formation (Figure 10B). The SFB25 binder showed significantly higher ALP activity than the others. In addition, the SFB50 binder had greater ALP activity than the others, except for the SFB25 binder. The ALP activity of the SFB100 and SFB0 binders was rather similar. The ALP activity of the SFB75 binder was significantly lower than the others on day 7. On day 14, the SFB25 binder still showed higher ALP activity than the others. The SFB75 binder obviously increased from day 7 to 14 and was higher than the SFB100, SFB50, and SFB0 binders. The SFB50 binder had much lower ALP activity than the others on day 14. The ALP activity of all binders decreased from day 14 to day 21; however, that of the SFB25 binder continued to be higher than the others. The SFB50 binder showed the lowest ALP activity on days 14 and 21.

Protein synthesis was measured on days 7, 14, and 21. Protein synthesis tended to increase from day 7 to day 14 and became lower on day 21 (Figure 10C). The SFB25 binder had significantly higher protein synthesis than the others, especially compared to the SFB100, SFB75, and SFB50 binders. The SFB25 binder had the highest protein synthesis, while the SFB100, SFB75, and SFB50 binders were lower. On day 21, all binders had lower protein synthesis; however, the SFB25 binder had the highest amount of protein synthesis, with statistical significance. The SFB75 binder had the lowest amount of protein synthesis.

## 4. Discussion

In our research, gelation of the binders was studied. Our results showed that SF binders were activated by incubation at 37 °C for 30 min, which matched body temperature. Interestingly, some of the previously published literature reported that fibril proteins acted as adhesive materials that solidified via self-assembly [45]. Normally, self-assembly is demonstrated by a sigmoid curve that follows three steps of assembly: (1) nucleation stage, (2) fibrillation stage, and (3) steady stage [46]. In the nucleation stage, some protein molecules act as seeders for attachment of other molecules. In the fibrillation stage, protein molecules attach to the seeders, which then propagate and organize into pre-mature fibrils. Self-assembly then approaches the steady stage when mature fibrils are formed.

**Figure 11:**
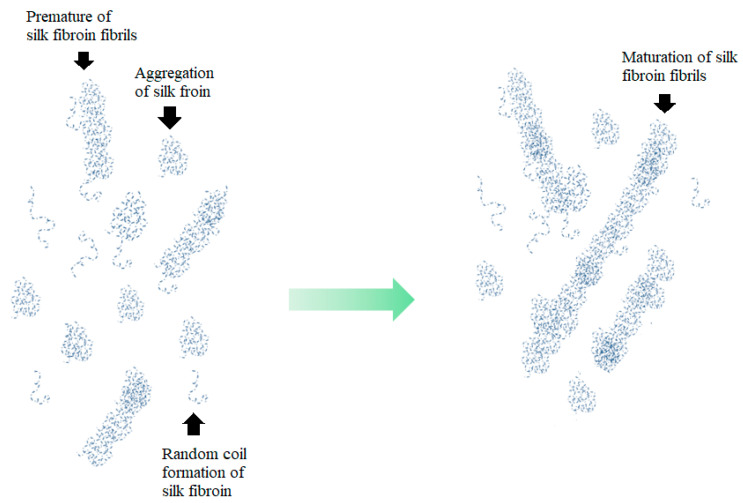
Proposed self-assembly of silk fibroin binder.

Our results showed that the SF binder exhibited self-assembly following those three steps of assembly. However, there were some differences between the fibril proteins reported in the literature and our binders. In our research, the binders had components of random coils, aggregation, and SF fibrils. The binders showed integrated self-assembly of these components. In the case of the SF100 binder, the self-assembly came from aggregated SF incorporated with random coils of SF. The SF75, SF50, and SF25 binders had combined self-assembly of aggregation, random coils, and premature SF fibrils. Finally, the SF0 demonstrated self-assembly of premature SF fibrils. Notably, our self-assembly of SF had more fluctuation in the sigmoid curve than the proteins reported in the literature because our binders had three components that interfered with each other during self-assembly monitoring. The proposed self-assembly of the silk fibroin binder is shown in Figure 11.

Molecular organization was related to gelation. The results showed that mixtures of random coils and SF aggregation with and without SF fibers had molecular organization via the amide groups. The mixtures of random coils and SF aggregation with SF fibers demonstrated stronger molecular interaction than without the SF fibers. This is related to the gelation of fibrin glue, which interacts via chemical groups for molecular organization [47]. Furthermore, our results showed that a mixture of random coils and SF aggregation had a lower melting temperature than SF fibers. Thus, the mixture of random coils and SF aggregation had lower structural stability than SF fibers; however, the mixtures of random coils and SF aggregation with SF fibers were more stable than without SF fibers. Therefore, the SF fibers showed the ability to enhance the structural stability of the binders.

Morphology is a clue that affects the performance of the binders [48]. In our research, the morphology after freeze-drying was observed as an indicator of the performance of the binders. The morphology showed that mixtures of random coils and SF aggregation with SF fibers had a hybrid morphology of the main pores with a fibril network. The main pores had some smooth walls, which is the nature of a mixture of random coils and SF aggregation. Some parts of the wall showed an SF fibril network distributed in the pores. Some fibrils were embedded into the texture of the wall, which caused the binders with SF fibers to have smaller pore sizes than the binders without SF fibers.

Previous research demonstrated that a porous structure provides suitable space for cell proliferation [49]. Importantly, the fibril network offers cell adhesion, which leads to enhanced cell proliferation [50]. Our results demonstrated that mixtures of random coils and SF aggregation with SF fibers had a hybridized morphology of a porous and fibril network structure that is suitable for enhancing cell adhesion and proliferation, which leads to promoting new tissue formation [51].

The topography observations of the binders after coating on glass slide showed that mixtures of random coils and SF aggregation with SF fibrils had greater roughness than without SF fibrils. Importantly, previous research showed that high surface roughness of materials promotes cell adhesion, spreading, migration, and proliferation [52]. Furthermore, a high surface roughness enhances the absorption of the biological signals secreted from cells [53]. This feature leads to inducing cell behaviors to promote tissue formation. Our mixtures of random coils and SF aggregation with SF fibrils showed a surface morphology that promises to enhance biological signals and cell behaviors.

Physical properties are an important guide in the design of binders with good performance. The contact angle is principally used to explain the surface characteristics of a material, especially when the contact angle is related to hydrophilicity, which is the most important property of a binder. The contact angle is used to elucidate biological-signal adsorption and cell behaviors at the interfacial area of the material and tissue [54]. One study demonstrated that materials with high hydrophilic materials had enhanced absorption of some biological signals secreted from cells during new tissue formation [55]. Another study showed that those materials promoted cell adhesion, spreading, migration, and proliferation [56]. In our research, the mixtures of random coils and SF aggregation with SF fibrils (SFB75, SFB50, and SFB25 binders) had higher hydrophilicity than the binder without SF fibers (SFB100) and the binder without a mixture of random coils and SF aggregation (SFB0). The mixtures of random coils and SF aggregation with SF fibrils are suitable to promote cell behaviors that lead to enhanced tissue formation [57], which is promising for bone formation.

SF release from a binder is a physical property that was tested in our research. SF was the main protein released from the binders that is related to the physical stability of a binder that, in turn, leads to cell behaviors. One study showed that a stable binder was suitable for tissue formation and promoted cell behaviors that led to enhanced bone formation. Our results showed that the mixtures of random coils and SF aggregation with SF fibrils had lower release than without SF fibrils. This demonstrated the physical stability of the mixtures of random coils and SF aggregation with SF fibrils, which was greater than without SF fibrils. Our binders showed performance that was in accordance with the literature and indicated that our binders had the physical stability to promote bone formation.

The mechanical properties of the binders were tested to assess the physical properties needed to maintain volume in a defect without collapse during bone augmentation. Our research demonstrated that the binders with SF fibrils had more mechanical stability of binding than without SF fibrils. The literature described the fibril component as reinforcement in a composite material [58]. Generally, composite materials have two components: matrix and reinforcement [59]. Our binders showed that a mixture of random coils and SF aggregation acted as the matrix, while the SF fibrils acted as the reinforcement. The SFB25 binder in this current research had the highest maximum load. The literature reported that a certain ratio of matrix to reinforcement will produce a good composite material [60]. Further, the matrix component will have good physical interaction with the reinforcement at a certain ratio [61]. The ratio of the mixture of random coils and SF aggregation to SF fibrils in the SFB25 binder had a good performance that followed the literature and had the physical characteristics that showed promise to promote bone healing. Nevertheless, hydrogel has the limitation of physical stability and mechanical strength in the long term, particularly in the wet stage [Figure S1], such as in a living organism. Therefore, enhancement of the physical stability and mechanical strength in the wet stage of our binder needs to be emphasized in future work.

Biological performance is the important clue for guidance to create binders for applications in bone healing. In our research, cell viability and proliferation, ALP activity, calcium deposition, and protein synthesis were selected as the biomarkers to indicate the biological performance of the binders. The results demonstrated that mixtures of random coils and SF aggregation with SF fibrils had better cell viability than without SF fibrils. Mixtures of random coils and SF aggregation with SF fibrils showed higher cell proliferation than without SF fibrils. Especially, the SFB25 binder had the greatest cell proliferation. This occurred because the SFB25 binder had suitable pore morphology with a fibril network for cell adhesion and proliferation.

Our results showed that the SFB25 binder had good ALP activity and calcium deposition, which indicated the biological function of osteoconduction. This occurred because the fibril network in the pores and the physical stability were suitable clues for the enhancement of osteoconduction [62]. Furthermore, the SFB25 binder had the highest level of protein synthesis, which relates to the secreted signals from cells leading to the enhancement of osteoconduction [63]. According to the biological performance results, the SFB25 binder had suitable properties for the fabrication of a promising binder for osteoconduction in bone repair.

**Figure 12:**
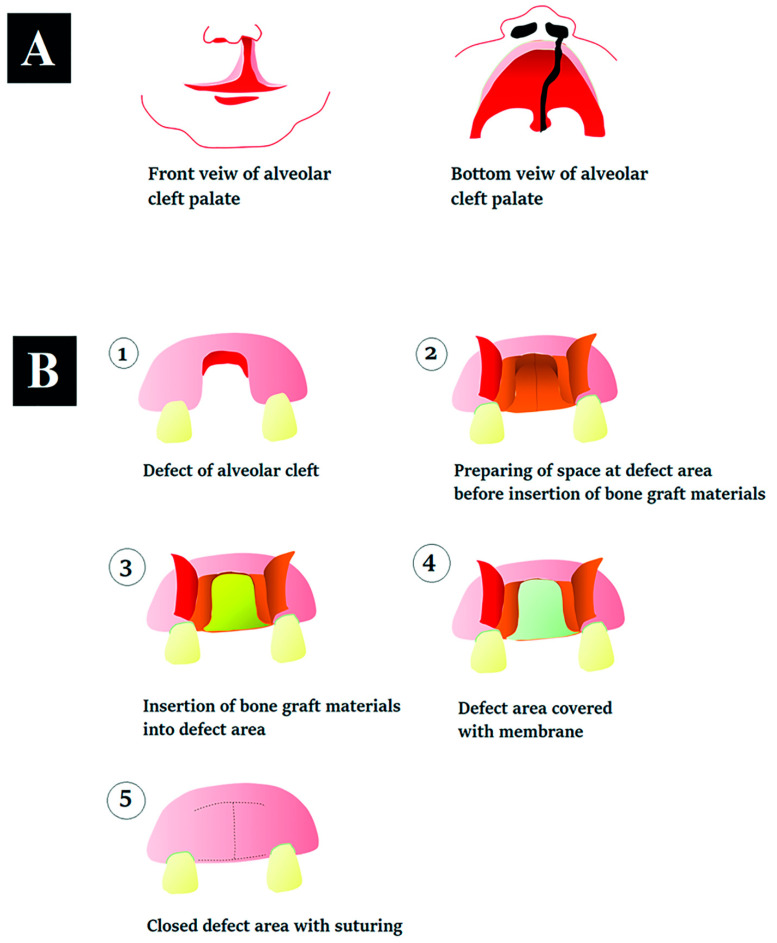
(**A**) Structure of an alveolar cleft palate; (**B**) none repair of an alveolar cleft palate with grafting materials.

An alveolar cleft palate is illustrated in Figure 12A, and the surgical procedure is shown in Figure 12B. It starts with preparation of a space at the defect with flapping. Then, the selected bone-grafting material is inserted into the space of the defect, before covering the space with a membrane followed by suturing to close the defect. According to the literature on the bone repair of an alveolar cleft palate, some glues are used with the bioactive powders to give stability to the graft [64,65]. However, glues are not stable during the process of bone formation [66]. Glues alone do not offer suitable biological functions for osteoconduction to enhance bone formation [67]. We propose our binders be mixed with a chosen ceramic powder following previous studies, before insertion into the defect space.

The results of our study demonstrated that the structural formation of our binders had suitable stability to support and promote bone formation. The binders in this research displayed favorable morphological properties of the fibril networks in the pores, which are able to promote cell behaviors leading to enhanced bone formation. Furthermore, our binders showed suitable physical properties and biological performance for bone formation. The SFB25 binder had the most suitable performance for applications in bone augmentation.

## 5. Conclusions

Osteoconductive binders based on SF were prepared by a mixture of random coils and aggregation of SF and SF fibrils that were fabricated for the bone repair of an alveolar cleft defect. The binders formed gelation via the self-assembly of the aggregation and fibrillation of the SF. The binders with SF fibrils showed more molecular interaction and structural stability than gel binders without SF fibrils. The binders with SF fibrils had smaller pore sizes than without SF fibrils. The binders with low amounts of SF fibrils (i.e., SF100, SF75, and SF50) showed lower hydrophilicity than the binders with high amounts of SF fibrils (i.e., SF25 and SF0). The SF100 exhibited higher SF release than the others. The binders with SF fibrils had more mechanical stability than the binder without SF fibrils. The binders with SF fibrils had good biological performance as demonstrated by better cell viability and calcium deposition as well as higher cell proliferation, ALP activity, and protein release than the binder without SF fibrils. Especially, the SF25 binder showed better biological performance than the others and also demonstrated good osteoconductive ability that showed promise for the bone repair of an alveolar cleft defect. Nevertheless, in vivo testing needs to be done in future work to confirm the potential of our binders. Furthermore, the binders should be modified or incorporated with biofunctional molecules, such as bone morphogenetic proteins, cytokines, or bioactive molecules, to promote bone formation and angiogenesis.

## Figures and Tables

**Table 1:** Binder groups in the experiments.

Binder Groups	Details
SFB100	Mixture of random coils and aggregation of silk fibroin/silk fibroin fibrils (100:0)
SFB75	Mixture of random coils and aggregation of silk fibroin/silk fibroin fibrils (75:25)
SFB50	Mixture of random coils and aggregation of silk fibroin/silk fibroin fibrils (50:50)
SFB25	Mixture of random coils and aggregation of silk fibroin/silk fibroin fibrils (25:75)
SFB0	Mixture of random coils and aggregation of silk fibroin/silk fibroin fibrils (0:100)

## Data Availability

The data on this research are available from the corresponding authors upon reasonable request.

## Supplementary Materials

The following supporting information can be downloaded at: https://www.mdpi.com/article/10.3390/jfb13020080/s1. Supplementary Material File (pdf) containing: Figure S1: The stability of the binding under wet conditions, the scale bar represents 1 cm.
