# Supplementary material for: Osteoconductive Silk Fibroin Binders for Bone Repair in Alveolar Cleft Palate: Fabrication, Structure, Properties, and In Vitro Testing
Source: J Funct Biomater. 2022 Jun 14;13(2):80. doi: 10.3390/jfb13020080 (PMC9224859; doi:10.3390/jfb13020080)
Supplement: Supplementary file 1 [file jfb-13-00080-s001.zip › jfb-1711127-supplementary.pdf]

Supplement results

A binder in a wet state for preparing specimens to test mechanical properties

Method

The 400 ul SF binding in all groups was dropped on the glass slide and covered with another glass slide. The sample was kept at 37 °C for 1 h and dried at room temperature. The stability of the binder was observed after dropping 1000 ul of PBS to the area of the binder at different time points.

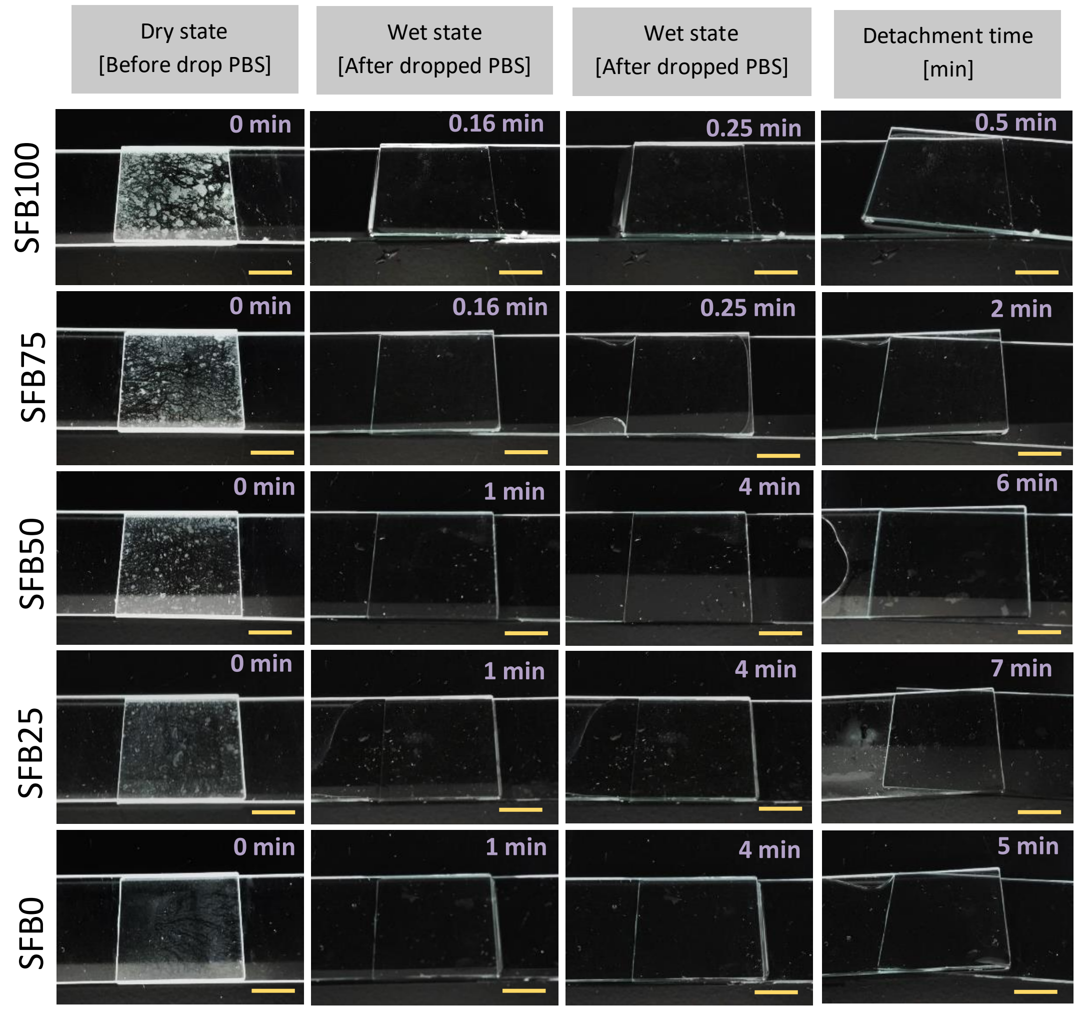

Supplement Figure S1. The stability of the binding under wet conditions, the scale bar represents 1 cm.

Result

After dropping SF binder, the results showed that SBF100 and SBF75 were easily detached in the first 10 and 15 seconds respectively. When the SF ratio increased, it had an effect on the stability of the SF binder. SBF25 had higher binder stability compared to other groups and SBF0 was greater than SBF100 and SBF75 but lower than SBF50. However, all samples were deformed after dropping PBS for few minutes. This is not suitable for testing mechanical properties.
